# Pyrotinib-based treatments in HER2-positive breast cancer patients with brain metastases

**DOI:** 10.1080/07853890.2022.2139411

**Published:** 2022-11-04

**Authors:** Xiaoping Ma, Yan Li, Li Li, Chunyan Gao, Dan Liu, Hongyu Li, Zhenhui Zhao, Bing Zhao

**Affiliations:** Breast Cancer, Xinjiang Medical University Affiliated Tumor Hospital, Urumqi, China

**Keywords:** Breast cancer, pyrotinib, trastuzumab, combination therapy, human epidermal growth factor receptor 2 (HER2), brain metastasis

## Abstract

**Objectives:**

Extensive application of anti-HER2 targeted therapy improves significantly the HER2-positive advanced breast cancer (BC) prognosis, however, it is still difficult to treat brain metastasis. In current study, we explored effective approaches *via* combining pyrotinib to treat brain metastasis in patients with HER2-positive advanced BC based upon clinical data.

**Materials and methods:**

Current study included 61 HER2-positive BC patients with brain metastases (BM) who were treated by pyrotinib-based regimens. The systemic regimens included pyrotinib combined with capecitabine, pyrotinib combined with nab-paclitaxel, and pyrotinib combined with vinorelbine. Patients’ progression-free survival (PFS), overall survival (OS), clinical benefit rate (CBR) and objective response rate (ORR), as well as drug-related adverse events (AEs) in regard of each combination regimen were analyzed.

**Results:**

Pyrotinib-based systemic therapy resulted in 8.6 months median PFS (mPFS) and 18.0 months median OS (mOS) among the BM patients. Regarding different regimens, the combination of pyrotinib with nab-paclitaxel was superior to the combination with capecitabine and vinorelbine with respect to PFS and OS. The central nervous system (CNS) ORR did not showcase significant difference among 3 regimens, however, nab-paclitaxel combined regimen obtained the best peripheral ORR (84.6%) (*p* ≤ .05).

**Conclusions:**

Pyrotinib-based combination therapy is safe for HER2-positive brain metastasis treatment. Compared with vinorelbine or capecitabine, pyrotinib combined with nab-paclitaxel is more effective with less toxicity, which is the preferable regimen for HER2-positive brain metastasis.

**KEY MESSAGES**
- Present investigation investigated effective methods through combining pyrotinib to treat brain metastasis with HER2-positive advanced brain cancer. The outcomes verified that pyrotinib-based combination therapy was safe and efficient to treat HER2-positive brain metastasis. Therefore, it is effective to treat brain metastasis applying anti-HER2 targeted therapies although pyrotinib showcases efficiency regarding its treatments for the metastasis.

## Introductions

Breast cancer (BC) has surpassed lung cancer to be the most popular malignant tumor in the world. There were 2.3 million BC cases that diagnosed in 2020, which accounted for 11.7% cancer patients [1]. In recent years, BC incidence and mortality in China have incremented significantly. In 2020, there were around 416,000 new cases along with 117,000 deaths in China [2]. Human epidermal growth factor receptor-2 (HER2) is a key oncogenic driver. HER2-positive BC results in 15%–20% all BC cases. This class of BC is highly aggressive, which has a poor prognosis [3]. With the application of anti-HER2-targeted drugs, the patient survival time in regards to HER2-positive BC has been improved significantly [4], though up to 50% brain metastasis rate is the most challenging issue [5].

There are no standard treatment approaches for brain metastases (BM) since current strategies generally include local therapy, e.g. surgery, whole brain and/or stereotactic radiation therapy and systemic therapy [6–8]. Anti-HER2-targeted therapy is of great significance for HER2-positive BM patients. Continuous anti-HER2 therapies may decrement mortality by around 50% [9]. Comparing with traditional macromolecular anti-HER2 monoclonal antibodies, tyrosine kinase inhibitors (TKIs) are superior to oral administration, multiple targets, as well as low cardiotoxicity [10]. Regarding the BM treatments, small molecule TKIs have been illustrated to owe better blood-brain barrier permeability along with definite anti-intracerebral tumor activity [11,12]. Pyrotinib is an oral, irreversible pan-HER2 receptor tyrosine kinase inhibitor targeting HER family such like HER1, HER2 and HER4 [13], which is approved for advanced BC treatments in China [14]. PHENIX investigation [15] showcased that among patients who took taxane and trastuzumab therapy, comparing with pyrotinib alone, pyrotinib combined with capecitabine could increment objective response rate (ORR) (68.6% vs. 16.0%, *p* < .001) and progression-free survival (PFS) (11.1 months vs. 4.1 months, *p* < .001). PFS was also prolonged in 30 BM patients (6.9 months vs. 4.2 months, *p* = .011) [16]. The above-mentioned studies have small sample sizes, which are lack of overall survival (OS) data. Some clinical challenges such as how to combine drugs for capecitabine-treated patients, whether to reuse chemotherapy after trastuzumab-resistant BM, whether radiotherapy is important for asymptomatic BM patients, etc. have not yet been addressed. Our study aimed to investigate BM HER2-positive patients and evaluate the safety along with efficacy of pyrotinib-based therapies.

## Materials and methods

### Ethics statement

Current investigation was a multi-center and retrospective clinical study. Patients came from multiple hospitals including Xinjiang Medical University Affiliated Cancer Hospital, Xinjiang Medical University Affiliated Traditional Chinese Medicine Hospital, Bazhou People’s Hospital, Hami Cancer Hospital, Yili Friendship Hospital, Dushanzi People’s Hospital, Aksu District People’s Hospital, etc. From 1 January 2019 to 31 May 2021, 61 HER2-positive patients who were treated with pyrotinib post BM were included in the study. Following-up prolonged until 31 December 2021. The 3rd Affiliated Teaching Hospital of Xinjiang Medical University (Affiliated Tumor Hospital) approved this study with approval no. [2018]05-228-06.

### Inclusion criteria

(1) Pathologically confirmed HER2-positive BC and measurable central nervous system (CNS) metastases (one or more brain parenchymal lesions with a diameter ≥10 mm), CNS progression after any CNS-targeted treatments (whole brain radiotherapy, stereotactic radiosurgery, surgery, systemic or combination treatment). (2) Eastern Cooperative Oncology Group (ECOG) performance score (PS) ≤2. (3) Expected survival time ≥3 months (4) Organ functions were basically normal. Blood routine, liver and kidney functions were basically normal, and there were no contraindications for treatments.

### Exclusion criteria

(1) Pregnant or breastfeeding. (2) Previously treated with pyrotinib. (3) Missing treatment information or accepted <2 cycles of pyrotinib treatment.

### Treatment and dosage adjustment

All patients received pyrotinib-based regimens with 21 days as a cycle. The initial dose, combined chemotherapies and/or targeted drugs, and local treatments such as radiotherapy and surgery (Table 1) were prescribed by physicians based upon previous clinical results, general health status and patient’s preference. Relevant data were collected in an electronic case report form. All patients provided written consent that informed.

**Table 1.** Patient characteristics at baseline.

Characteristics	No. (%) (*n* = 61)
**Age, median (range, year)**	53.5 (32 ∼ 68)
HR status	
HR positive	28 (45.9%)
HR negative	33 (54.1%)
ECOG performance score	
0	21 (34.4%)
1	36 (59.0%)
2	4 (6.6%%)
Metastatic sites	
Brain	61 (100%)
Lymph nodes	41 (67.2%)
Lung	28 (45.9%)
Pancreas	1 (1.6%)
Liver	27 (44.3%)
Bone	31 (50.8%)
Pleura	18 (29.5%)
Local recurrence	6 (9.8%)
Contralateral breast	1 (1.6%)
No. of metastatic sites	
1	5 (8.2%)
2	14 (23.0%)
3	22 (36.1%)
≥4	20 (32.7%)
Visceral metastases	
YES	41 (67.2%)
NO	20 (32.8%)
PriorHER2-targeted therapy	
Trastuzumab	59 (96.7%)
Lapatinib	24 (39.3%)
T-DM1	1 (1.6%)
Pertuzumab	5 (8.2%)
Local CNS therapy	
Surgery	1 (1.6%)
SRS	2 (3.2%)
WBRT	44 (72.2%)
Two or more prior	3 (4.9%)
NO	11 (18.1%)

### Evaluation criteria

The primary endpoints were PFS and OS, which were defined as the time from initial pyrotinib treatment to disease progression that confirmed by CT/MRI and/or death for any reasons. Primary and secondary endpoints included ORR, CBR, OS and safety. ORR is the proportion of patients with complete response (CR) or partial response (PR) (ORR = CR + PR). Clinical benefit rate (CBR) is the proportion of patients with CR, PR and stable disease (SD) (CBR = CR + PR + SD). OS is defined as the time from initial pyrotinib treatment to death for any causes, or, the last follow-up. The severity of adverse reactions (grade 0–4) was determined according to National Cancer Institute-Common Terminology Criteria for Adverse Events version 4.0 (NCI-CTC v4.0).

### Statistical analyses

According to NCCN and CSCO guidelines, targeted therapy combined with single chemotherapy is recommended for second-line or later line treatment of HER-positive advanced BC. The chemotherapies that recommended included capecitabine, taxanes and vinorelbine. Based upon patient previous treatment histories, we divided 61 patients into 3 groups: pyrotinib plus capecitabine group (*n* = 35, 57.4%), pyrotinib plus nab-paclitaxel group (*n* = 17, 27.9%) and pyrotinib plus vinorelbine group (*n* = 9, 14.7%). Differences of categorical features among the 3 groups were examined *via* Pearson’s χ^2^ or Fisher’s exact test. Nonparametric Mann-Whitney U test was employed for comparison of continuous features not meeting normal distribution. Log-rank test was used for comparisons of PFS estimated by Kaplan-Meier method among groups. Univariable and multivariable Cox proportional hazard model was employed to assess the covariate effects on PFS. Statistical analyses were performed by using SPSS 25.0 software. Descriptive statistics were utilized to analyze clinical characteristics among patients. *p* < .05 was considered as statistically significant. Survival analysis and visualization were performed on R platform (version 4.0.4, https://www.r-project.org/).

## Results

### Baseline characteristics

Clinical data with respect to 61 patients who received pyrotinib-based treatment from 1, January 2019 to 31 May 2021 were analyzed. Detailed information of baseline characteristics was listed in Table 1. The median age of patients was 54 years old (32 ∼ 68 years old). There were 28 patients (45.9%) who were hormone receptor-positive, 56 patients (91.8%) who had BM and/or other organ metastases, and 42 patients (68.8%) had ≥3 metastatic sites. In terms of targeted therapy, 59 patients (96.7%) were treated with trastuzumab, 24 patients (39.3%) were treated with lapatinib, 5 patients (8.2%) were treated with pertuzumab, and 1 patient (1.6%) was treated with T-DM1. 50 patients received CNS local treatment including 1 case surgery (1.6%), 2 cases SRS (3.2%), 44 cases WBRT (72.2%), and 3 cases receiving ≥2 local treatments (4.9%). There were 11 patients who did not receive any local treatments (18.1%).

### Treatment and dosage adjustment

54 (88.6%) patients were initially treated with a standard dose of 400 mg/d of pyrotinib, and 7 (11.6%) patients were initially given a reduced dose. During the whole treatment process, 15 patients underwent dose reduction treatment, most of which were 400 mg/*d*→320 mg/d (86.7%) (Table 2).

**Table 2.** Treatment administration.

Pyrotinib treatment	Patients (*N* = 61)
**Lines of pyrotinib-based therapy for BC brain metastatic**	***N* (%)**
1	10 (16.4%)
2	26 (42.6%)
3	18 (29.5%)
≥4	7 (11.5%)
**Starting dose of pyrotinib (mg/day), *n* (%)**	
400	54 (88.6%)
320	6 (9.8%)
240	1 (1.6%)
**Regimens**	***N* (%)**
Regimen with capecitabine	35 (57.4%)
Pyrotinib plus capecitabine	29 (47.6%)
Pyrotinib plus capecitabine plus trastuzumab	6 (9.8%)
Regimen with Nab-paclitaxel	17 (27.9%)
Pyrotinib plus Nab-paclitaxe	15 (24.6%)
Pyrotinib plus Nab-paclitaxe plus trastuzumab	2 (3.2%)
Pyrotinib with Vinorelbine	
Pyrotinib plus Vinorelbine	9 (14.7%)

### Efficacy evaluation

**Figure 1.**
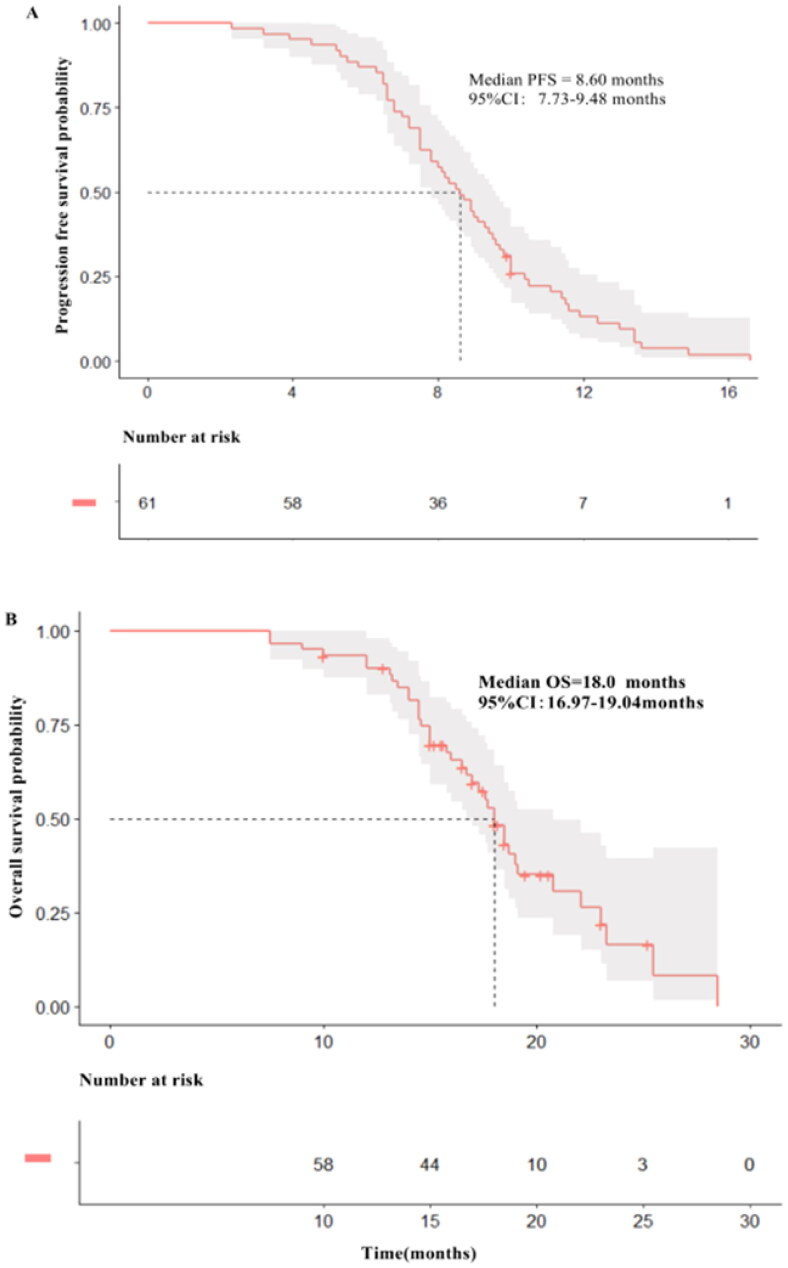
(A) Kaplan-Meier estimates of progression-free survival (PFS) for all patients treated with pyrotinib-based therapy (B) Kaplan-Meier estimates of over survival (OS) for all patients treated with pyrotinib-based therapy.

The median follow-up time was 18.5 months, and events reaching PFS and OS were 59 (96.7%) and 37 (60.7%). The median PFS was 8.6 months (95% CI: 7.73–9.48) (Figure 1(A)) and median overall survival was 18.0 months (95% CI: 16.97–19.04) (Figure 1(B)).

### Survival analysis of different chemotherapeutics

**Figure 2.**
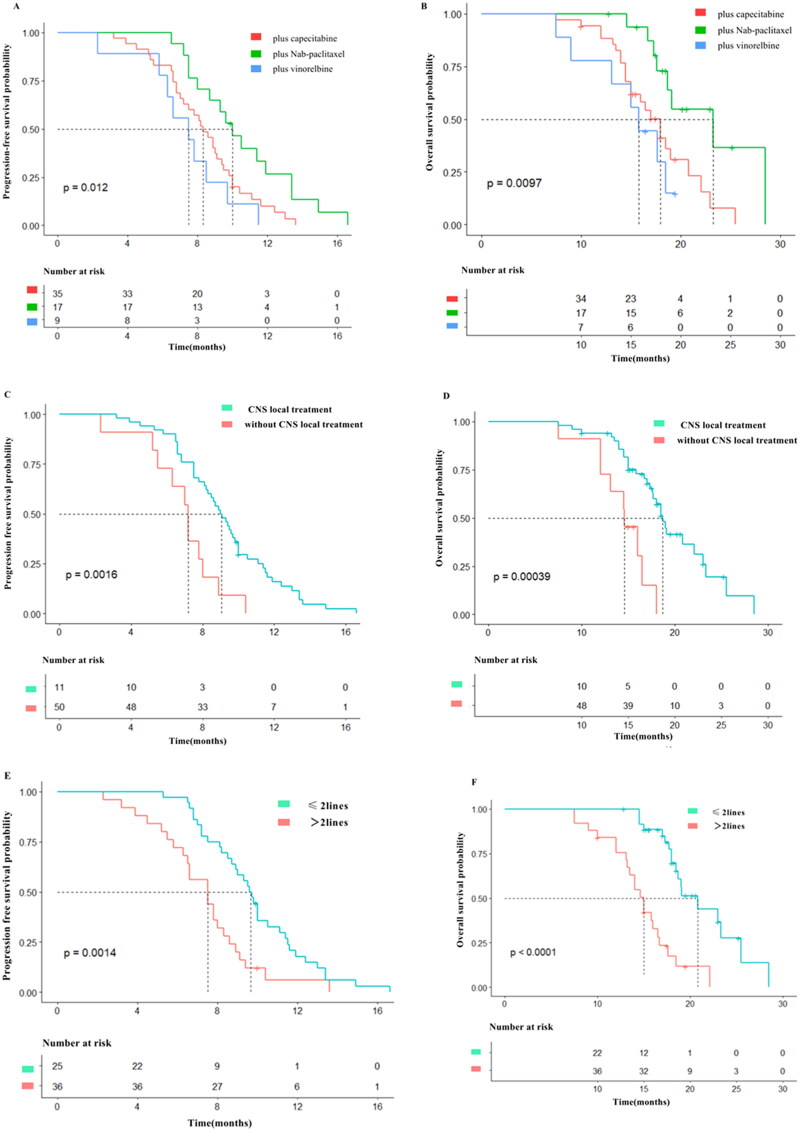
Kaplan-Meier estimates of progression-free survival (PFS) and over survival (OS) for patients stratified by treatment regimens. (A) PFS according to pyrotinib plus Nab-paclitaxel (median PFS = 10.0 months; 95% CI:8.49–11.51 months), pyrotinib plus capecitabine (median PFS = 8.3 months; 95% CI:7.24–9.36 months), pyrotinib plus vinorelbine (median PFS = 7.5 months; 95% CI:4.87–10.13 months). (B) OS according to pyrotinib plus Nab-paclitaxel (median OS = 23.3 months; 95% CI:17.49–29.11 months), pyrotinib plus capecitabine (median OS = 18.0 months; 95% CI:15.81–20.19 months), pyrotinib plus vinorelbine (median OS = 15.8 months; 95% CI:13.46–18.14 months). (C) PFS according to with CNS local treatment (median PFS = 9.0 months; 95% CI:8.07–9.92 months), PFS according to without CNS local treatment (median PFS = 7.2 months; 95% CI:6.26–8.14 months). (D) OS according to with CNS local treatment (median OS = 18.7 months; 95% CI:17.43–19.96 months), OS according to without CNS local treatment (median OS = 14.6 months; 95% CI:12.04–17.16 months). (E) PFS according to ≤ 2 lines (median PFS = 9.6 months; 95% CI:8.86–10.34 months), PFS according to >2 lines (median PFS = 7.5 months; 95% CI:6.04–8.96 months). (F) OS according to ≤ 2 lines (median OS = 18.7 months; 95% CI:17.43–19.96 months), OS according to >2 lines (median OS = 14.6 months; 95% CI:12.04–17.16 months).

In this study, 61 patients were divided into pyrotinib plus capecitabine group (*n* = 35, 57.4%), pyrotinib plus nab-paclitaxel group (*n* = 17, 27.9%), and pyrotinib plus vinorelbine group (*n* = 9, 14.7%). Pairwise comparison regarding PFS and OS showcased that there were significant differences in PFS and OS between pyrotinib plus nab-paclitaxel group and pyrotinib plus capecitabine group (10.0 vs. 8.3 months for PFS, 23.3 vs. 18.0 months for OS, *p* < .0167 for both). Pyrotinib plus nab-paclitaxel group was with a significant longer PFS and OS comparing with pyrotinib plus vinorelbine group (10.0 vs. 7.5 months for PFS, 23.3 vs. 15.8 months for OS, *p* < .0167 for both). However, PFS and OS in pyrotinib plus capecitabine group and pyrotinib plus vinorelbine group did not give significant differences (8.3 vs. 7.5 months for PFS, *p* = .229; 18.0 vs. 15.8 months for OS, *p* = .331). Data showcase that for HER2-positive BC patients with BM, nab-paclitaxel-containing regimen is superior to capecitabine-containing or vinorelbine-containing regimen regarding PFS and OS (Figure 2(A and B)).

### Survival analysis regarding CNS local treatment

Among the 61 patients that included, 50 received CNS local treatment including 1 case of surgery (1.6%), 2 cases of SRS (3.2%), and 44 cases of WBRT (72.2%). 3 cases received two or more CNS local treatments (4.9%). 11 cases (18.1%) did not receive any CNS local treatments. Patients were divided into 2 sub-groups according to whether receiving CNS local treatment. There were significant differences in PFS and OS between local treatment group and no local treatment group (9.0 vs. 7.2 months for PFS, *p* < .001; 18.7 vs. 14.6 months for OS, *p* < .001) (Figure 2(C, D)), advising that CNS local treatment can improve PFS and OS in terms of patient population.

### Survival analysis of pyrotinib-containing regimens as different line treatment

In present study, 10 patients (16.4%) received pyrotinib-containing regimen as first-line treatment, and 26 cases (42.6%) as the second-line, 18 cases (29.5%) with third-line treatment and 7 cases (11.5%) with ≥ fourth-line treatment. PFS and OS were better for patients receiving treatment as ≤ second-line treatment than those receiving treatment as > second-line treatment (9.6 vs. 7.5 months for PFS, *p* = .001; 20.8 vs. 15.0 months for OS, *p* = .001). The data suggest that HER2-positive BC patients with BM should be treated early with pyrotinib-based regimen (Figure 2(E and F)).

### Intracranial and peripheral ORR

Among 61 BM patients, overall intracranial ORR was 39.3%. The ORR was 37.1% for capecitabine group, 47.1% for nab-paclitaxel group, and 33.3% for vinorelbine group. Chi-square test did not give significant difference in intracranial ORR among the 3 groups.

**Figure 3.**
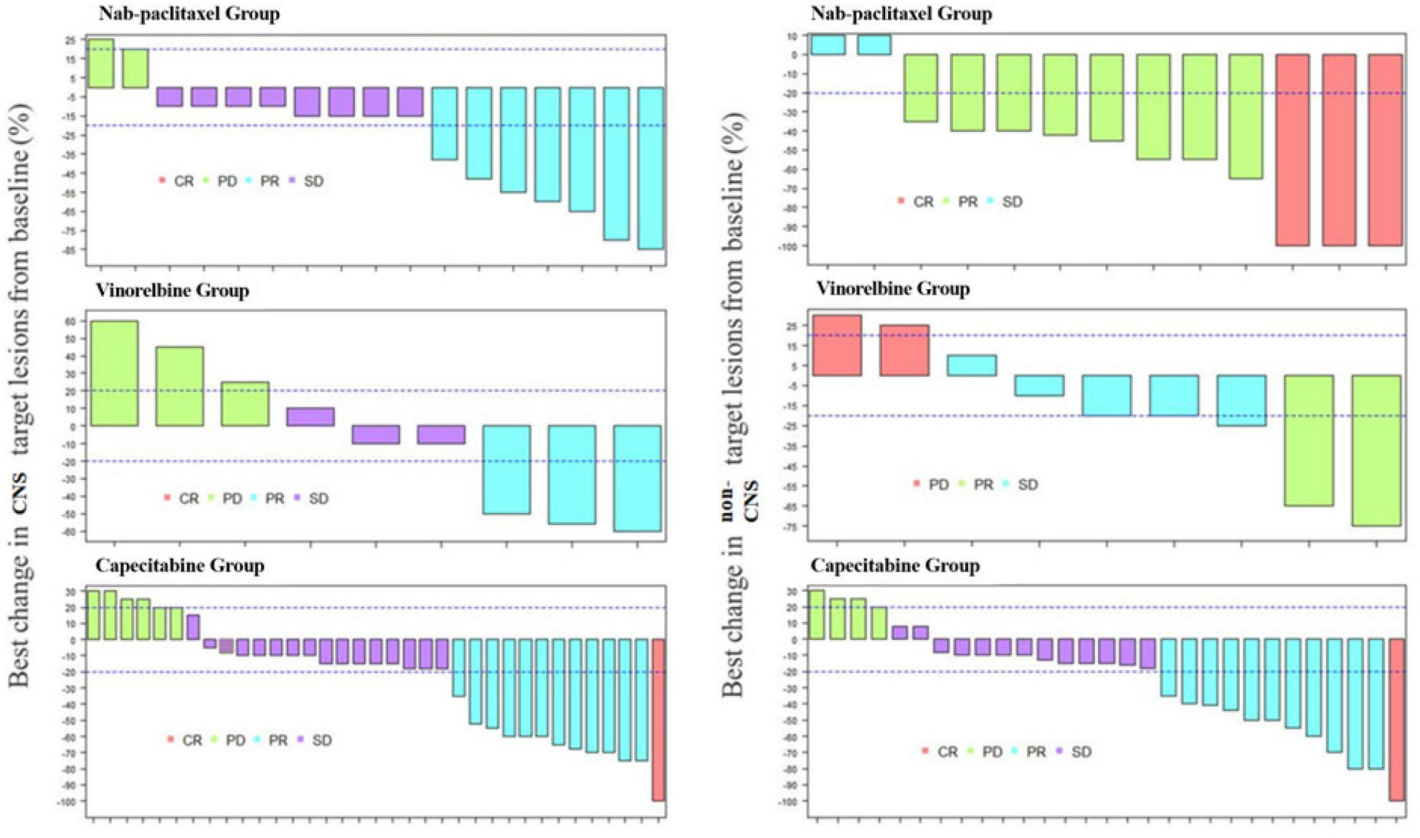
Waterfall plot for best changes in CNS and non-CNS target lesions from baseline. Dashed lines at 20% and –30% denote thresholds for progressive disease and partial response respectively, according to the Response Evaluation Criteria In Solid Tumours, version 1.1.

Among the 51 patients with peripheral measurable lesions, the overall peripheral ORR was 49.0%, and that was 41.4% for capecitabine group, 84.6% for nab-paclitaxel group, and 22.2% for vinorelbine group. There was significant difference in peripheral ORR among the 3 groups (*p* = .006). After adjusting *p* values by the Bonferroni method, further pairwise comparisons showcased that peripheral ORR in nab-paclitaxel group was better than that in capecitabine group (*p* = .0346) as well as vinorelbine group (*p* = .0219) (Table 3 and Figure 3).

**Table 3.** Best regimen response.

	Capecitabine Group	Nab-paclitaxel Group	Vinorelbine Group
Best CNS response	35	17	9
Complete response	1 (2.9%)	0	0
Partial response	12 (34.3%)	8 (47.1%)	3 (33.3%)
Stable disease	15 (42.8%)	7 (41.1%)	3 (33.3%)
Progressive disease	7 (20.0%)	2 (11.8%)	3 (33.4%)
Objective response rate	13 (37.1%)	8 (47.1%)	3 (33.3%)
Disease control rate	28 (80.0%)	15 (88.2%)	6 (66.6%)
Best non-CNS response	29	13	9
Complete response	1 (3.5%)	3 (23.1%)	0
Partial response	11 (37.9%)	8 (61.5%)	2 (22.2%)
Stable disease	13 (44.8%)	2 (15.4%)	5 (55.6%)
Progressive disease	4 (13.8%）	0	2 (22.2%)
Objective response rate	12 (41.4%)	11 (84.6%%)	2 (22.2%)
Disease control rate	25 (69.0%)	13 (100%)	7 (77.8%)

### Toxicity and adverse events (AEs)

All grade AEs were recorded. The most common AE was diarrhea, which was reported among 52 patients (85.2%), and ≥3 grade diarrhea was reported in 12 patients (19.7%). ≥3 grade AEs also included 3 neutropenia cases (4.8%), 2 cases of leukopenia (3.2%), 2 nausea and vomiting cases (3.2%). Each case had hand-foot syndrome, thrombocytopenia, anemia, fatigue, neurological toxicity and weight loss.

Regarding combined chemotherapies, diarrhea incidences in capecitabine group, nab-paclitaxel group and vinorelbine group were 34 cases (97.1%), 12 cases (70.6%) and 6 cases (66.7%), and incidences of ≥3 grade diarrhea in the above 3 groups were 8 cases (22.9%), 2 cases (11.8%) and 2 cases (22.2%) respectively. There were 15 cases of hand-foot syndrome in capecitabine group (42.9%), which did not occur in the other two groups. Nausea/vomiting incidences in the above three groups were 26 cases (74.3%), 5 cases (29.4%) and 2 cases (22.2%) respectively. Chi-square test showed only diarrhea (*p* = .006), hand-foot syndrome (*p* < .001), nausea/vomiting (*p* = .001) were significantly different among the three groups. After adjusting *p* values by Bonferroni method, pairwise comparison showed that the diarrhea incidence in nab-paclitaxel group was lower than that in capecitabine group (*p* = .034), and the incidence of hand-foot syndrome in the nab-paclitaxel group was lower than that in capecitabine group (*p* = .003). The nausea/vomiting incidence in both nab-paclitaxel group and vinorelbine group was lower than that in capecitabine group (*p* = .016 and *p* = .020 respectively) (Table 4).

**Table 4.** Grade 3 to 4 adverse events.

	Capecitabine Group (*n* = 35)		Nab-paclitaxel Group (*n* = 17)		Vinorelbine Group (*n* = 9)	
Adverse Event	Any grade	Grade ≥ 3	Any grade	Grade ≥ 3	Any grade	Grade ≥ 3
Diarrhea	34	8	12	2	6	2
Neutropenia	10	2	3	0	2	1
Hand-foot syndrome	15	1	0	0	0	0
Leukopenia	9	2	2	0	2	0
Anemia	5	1	2	0	2	0
Thrombocytopenia	11	1	2	0	4	1
Nausea and vomiting	26	3	5	0	2	0
Fatigue	5	1	5	0	1	0
ALT/AST increased	4	0	1	0	1	0
Rash	2	0	0	0	0	0
Neurotoxicity	8	1	9	1	2	0
Weight loss	9	1	3	0	2	0

## Discussions

With innovative targeted therapy emergence, BM treatment has begun to shift from local therapy to systemic therapy [17]. Anti-HER2-based combination therapy is the most important systemic therapy within the population [18]. However, the intracranial effect of monoclonal antibodies is limited due to their inefficiency to cross the blood-brain barrier [19,20]. Small molecule TKIs have showcased perfect efficacy in treatment of advanced BC including BM [11,21,22]. TKIs have mechanisms of action and the target domain are different to those of trastuzumab, which enabled them to be able to overcome trastuzumab resistance [23]. Trastuzumab-resistant patients can still benefit from TKI-containing regimens. Based upon the above reasons, as well as good accessibility, pyrotinib has been widely applied among Chinese patients with HER2-positive advanced BC such as BM [10].

In our study, 96.7% patients received trastuzumab only once. The mPFS of the patients receiving pyrotinib-based combination treatment was 8.6 months and the mOS was 18.0 months. Comparing with the HER2CLIMB study [24], mPFS was slightly lower (8.6 months vs. 9.9 months) though mOS was quite comparable. One possible reason is that the experimental groups in HER2CLIMB are all tucatinib plus trastuzumab dual-target combination regimen, while the proportion of pyrotinib plus trastuzumab dual-target in our study was only 16%. Encouragingly, mPFS in our study was >6.9 months in BM subgroup regarding the PHENIX study [12]. Our study is among the few studies to obtain OS data for BM, and the 18.0 months of mOS in our study was also better than the 13.93 months of mOS in another clinical study conducted by Anwar et al. [25]. The sample size of Anwar, M.’s study was relatively small (39 cases), and only 5 cases (12.8%) were given pyrotinib plus nab-paclitaxel regimen in comparison to 17 cases (27.9%) in our study. While the best PFS and OS were just obtained in patients being given nab-paclitaxel-contained regimen, which improved the overall efficacy in current study. In addition, M. Anwar’s study did not analyze the survival in BM by different combination regimens, and our study fills this gap in this regard.

Our study also provides additional therapeutic experience beyond clinical trials for the treatment of HER2-positive BM patients. Capecitabine, a chemotherapeutic drug commonly used in clinical practice, can be uptaken in BM [26]. In clinical trial, many patients often received capecitabine treatment in prior to pyrotinib. However, the combined effect of pyrotinib and other chemotherapeutic drugs is still unknown, and there are few reports regarding combinational pyrotinib application in BM.

Comparing with solvent-based paclitaxel, nab-paclitaxel can significantly improve the metastatic breast cancer (MBC) treatment efficacy [27]. One investigation advised that nab-paclitaxel combined with trastuzumab as first-line treatment of MBC showcased efficacy for BM [28]. In BM later-line treatment, nab-paclitaxel combined with trastuzumab was also a superb effective approach [29]. Vinorelbine has also demonstrated efficacy and tolerability in combination with trastuzumab and lapatinib [30,31]. These findings provide a rationale for the use of chemotherapeutic agents in combination with other anti-HER2 agents such like pyrotinib. In our study, pyrotinib combined with nab-paclitaxel had the best PFS (10.0 months), which was comparable with 9.9 months in HER2CLIMB study and better than 6.9 months in the PHENIX study. This may result from the advantage of nab-paclitaxel in peripheral ORR and good tolerability. Further analysis confirmed the benefit. In patients with only peripheral progression after the treatment of pyrotinib plus capecitabine, replacing capecitabine with nab-paclitaxel could also obtain 3.6-month PFS. The PFS in the pyrotinib plus vinorelbine group was 7.5 (4.87–10.13) months, in line with the result of a multicenter retrospective study by Yi Li et al. [32]. In present study, 28 hormone receptor-positive patients (45.9%) were not treated with pyrotinib plus endocrine therapy. A possible reason is that the intracranial efficacy of SERDs is still unclear [33], and although CD4/6 inhibitors have significant extracranial activity, the intracranial response rate is usually low [34,35].

Different from the data in Munawar Anwar et al. [25], our study showcased that the pyrotinib-containing regimen was ≤ 2 line treatment could achieve significantly better PFS than that of those with >2 line treatment (9.6 vs 7.5 months, *p* = .001), advising for HER2-positive BC patients with BM, pyrotinib-containing treatment should be given early.

In present study, 11 BM patients who received only pyrotinib-based therapy and were not given local control (surgery/radiotherapy) had 27.3% ORR, which was better than neratinib plus capecitabine regimen in TBCRC022 trial, but much lower than 74.6% of CNS ORR in recent PERMEATE study [36]. Despite that, ours and PERMEATE study illustrated the pyrotinib superiority in CNS control rate. The key is how to translate such ORR benefit to longer OS and PFS. Our study demonstrated that the patients receiving local treatment had a significant better PFS (9.0 vs. 7.2 months, *p* < .001) and OS (18.7 vs. 14.6 months, *p* < .001) than those without local treatment. For patients receiving local treatments, their PFS (9.0 months) had been close to the PFS of the Cohort A (11.3 months) in PERMEATE study. It should be noted that the PERMEATE study did not set up a control group, and the study included more patients without peripheral lesions or with unmeasurable peripheral lesions (54.2%). That is, there are more patients with pure BM or only bone metastases. Such patients only accounted for 16.4% cases in our study. Therefore, we proposed a combination treatment regimen for HER2-positive patients with BM. Unfortunately, due to small sample size, the specific effects, and potential mechanisms of pyrotinib in the sensitivity to radiotherapy/surgery needs to be further investigated, which is one limitation of this study. One possible explanation is that radiotherapy alters the permeability of the blood-brain barrier to increase the drug amount entering the brain [37], or to be related to the sensitization effect of pyrotinib on radiotherapy [38].

As to adverse reactions, diarrhea and vomiting were the most common grade 3–4 adverse reactions in current study, which is consistent with previous results [15,16]. Notably, the pyrotinib plus capecitabine group had more and more serious gastrointestinal reactions (vomiting, diarrhea) than the nab-paclitaxel group. Although all adverse events were effectively controlled by symptomatic drugs that did not lead to discontinuation, the changes of plasma concentration or metabolic behavior of anti-cancer drugs resulted from the interaction with symptomatic drugs may affect efficacy and safety of anti-cancer drugs [39]. It has been reported that the bioavailability of pyrotinib was decremented by 50.3% while using montmorillonite powder for antidiarrheal [40]. Hormonal drugs are usually used to prevent infusion reactions and assist with anti-vomiting, but they can also alter the activity of drugs metabolized by CYP3A4 enzymatic pathway [41]. While the pyrotinib metabolism in human body is mainly catalyzed by CYP3A4 [42]. CYP3A4 expression was not detected in present study, while a recent pharmacokinetic study demonstrated significant increase regarding pyrotinib exposure when pyrotinib was combined with CYP3A4 inhibitor itraconazole [39]. Concomitant use with CYP3A4 inducer rifampicin significantly reduced pyrotinib exposure [43]. Therefore, risky interaction between pyrotinib and potential CYP3A inhibitors or inducers should be assessed in clinics.

In our study, the combination of pyrotinib and nab-paclitaxel largely avoided the above risks, which reduced the use of antidiarrheal agents such as montmorillonite powder and hormonal drugs due to lower incidence of diarrhea and vomiting. In addition, compared with solvent-based paclitaxel, albumin-bound paclitaxel does not require solvents such as castor oil and ethanol, which avoids allergic reactions [44] and reduces the application of hormones. Also, albumin-bound nanoparticles can enrich the chemotherapeutic drugs in tumor tissue through the interaction with drug transport receptors, thereby increasing the antitumor activity [45]. In this manner, the combination of pyrotinib and nab-paclitaxel has brought unexpected results of ‘attenuating and synergistic’ to this group of patients.

There are some limitations of our investigations, however. Firstly, this study was a retrospective rather than prospective study, and there existed selection and information bias. Secondly, as mentioned above, the sample size in the study was relatively small and large-scale clinical studies are demanded to validate our findings. Thirdly, the use of pertuzumab and T-DM1 in our cohort was low, which was possibly due to accessibility or cost factors.

In conclusions, in the BM treatment regarding HER2-positive BC, effective systemic therapy can control both peripheral and intracranial metastases. Many drugs can be combined with pyrotinib, and how to choose an effective and appropriate regimen is critical. Comparing with vinorelbine or capecitabine, pyrotinib combined with nab-paclitaxel has the advantage of ‘low toxicity and high efficacy’, which is the preferable regimen for HER2-positive BM. Local therapy post BM is still recommended, in which the timing or intervention mode of radiotherapy (sequential or concurrent) needs to be clarified.

## Data Availability

All data are included in the manuscript. Upon requests to corresponding author, additional data would be available.
